# Critical steps in clinical shotgun metagenomics for the concomitant detection and typing of microbial pathogens

**DOI:** 10.1038/s41598-018-31873-w

**Published:** 2018-09-13

**Authors:** Natacha Couto, Leonard Schuele, Erwin C. Raangs, Miguel P. Machado, Catarina I. Mendes, Tiago F. Jesus, Monika Chlebowicz, Sigrid Rosema, Mário Ramirez, João A. Carriço, Ingo B. Autenrieth, Alex W. Friedrich, Silke Peter, John W. Rossen

**Affiliations:** 1 University of Groningen, University Medical Center Groningen, Department of Medical Microbiology, Groningen, The Netherlands; 2 Institute of Medical Microbiology and Hygiene, University of Tübingen, Tübingen, Germany; 3 Instituto de Microbiologia, Instituto de Medicina Molecular, Faculdade de Medicina, Universidade de Lisboa, Lisbon, Portugal

## Abstract

High throughput sequencing has been proposed as a one-stop solution for diagnostics and molecular typing directly from patient samples, allowing timely and appropriate implementation of measures for treatment, infection prevention and control. However, it is unclear how the variety of available methods impacts the end results. We applied shotgun metagenomics on diverse types of patient samples using three different methods to deplete human DNA prior to DNA extraction. Libraries were prepared and sequenced with Illumina chemistry. Data was analyzed using methods likely to be available in clinical microbiology laboratories using genomics. The results of microbial identification were compared to standard culture-based microbiological methods. On average, 75% of the reads corresponded to human DNA, being a major determinant in the analysis outcome. None of the kits was clearly superior suggesting that the initial ratio between host and microbial DNA or other sample characteristics were the major determinants of the proportion of microbial reads. Most pathogens identified by culture were also identified through metagenomics, but substantial differences were noted between the taxonomic classification tools. In two cases the high number of human reads resulted in insufficient sequencing depth of bacterial DNA for identification. In three samples, we could infer the probable multilocus sequence type of the most abundant species. The tools and databases used for taxonomic classification and antimicrobial resistance identification had a key impact on the results, recommending that efforts need to be aimed at standardization of the analysis methods if metagenomics is to be used routinely in clinical microbiology.

## Introduction

Classical microbial culture is still considered the gold standard in medical microbiology. Several molecular detection techniques have been implemented but these are generally geared towards specific pathogens (e.g. specific RT-PCR or microarrays). Even when unbiased molecular approaches are used, such as 16 S/18 S rRNA gene sequencing, these do not provide all the information that can be obtained by culturing, e.g., antimicrobial susceptibility and molecular typing information. However, microbial culture is laborious and time-consuming and new methods are needed to replace it. Ideally, a single method should provide rapid identification and characterization of clinically relevant pathogens directly from a sample in order to guide therapy, predict potential treatment failures and to reveal possible transmission events.

Shotgun metagenomics (SMg) is a culture-independent technique that provides valuable information not only at the identification level, but also at the level of molecular characterization. Studies have shown that it has added value in terms of detection sensitivity and personalized treatment in clinical microbiology, when identifying bacteria^1,2^ or viruses^3^. Indeed Gyarmati *et al*., 2016^4^, used a sequence-based metagenomics approach directly from blood to detect non-culturable, difficult-to-culture and non-bacterial pathogens. The authors were able, through SMg, to detect viral and fungal pathogens together with bacteria, which had not been detected through classical microbiology. Additionally, SMg can be used for infection prevention, having the potential to identify transmission events directly from clinical samples^5^. For example, SMg was proven valuable for the identification of inter-host nucleotide variations occurring after direct transmission of noroviruses causing gastroenteritis^5^. Hasman and colleagues (2014)^1^ were able to identify urinary pathogens directly from urine, as well as antimicrobial resistant genes compatible with the resistant phenotype determined through antimicrobial susceptibility testing. They also identified almost perfect phylogenetic matches between whole-genome sequence (WGS) data obtained by metagenomics and WGS of pure isolates.

Despite the promise of SMg of becoming a one-stop solution in clinical microbiology, SMg still has several challenges to overcome. One of the greatest challenges is the choice of the extraction and sequencing protocols, as well of the type of controls^6^. The extraction protocol should efficiently and specifically isolate microbial DNA/RNA, while removing the host DNA/RNA^7^. However, the variety of clinical samples used in the diagnosis of distinct types of infection (e.g. tissues versus fluids), poses a serious challenge for standardization, an essential step if these methods are to be used by routine diagnostic laboratories. The sequencing protocol is also dependent on the pathogens of interest (e.g. bacteria versus viruses), sequencing strategy (DNA and/or RNA), required turnaround time, sequencing depth and error tolerance^6^. The use of defined controls is necessary for validation of each experiment and these should be adapted for every type of infection and sample type and should consist of a combination of known positive specimens, pathogen-negative patient specimens and pathogen-negative patient specimens spiked with live microorganisms or pure DNA^6^.

Another potential challenge are the metagenomics analysis tools. Recent studies have evaluated the different SMg sequence classification methods^8^. These use different methodologies for classification: sequence similarity-based methods, sequence composition-based methods and hybrid methods^8^. They differ not only in the algorithms for detecting the microorganisms present, but also in the databases used. This high variability leads to different results, not only at the microorganism classification level but also when evaluating the relative abundance of these pathogens^8^. A recent study evaluated the accuracy of 38 bioinformatics methods using both *in silico* and *in vitro* generated mock bacterial communities. Dozens to hundreds of species were falsely predicted by the most popular software, and no software clearly outperformed the others^8^. In the absence of studies comparing the outputs of different analysis methods in clinical samples, users may decide which methods to use based on personal experience with a given tool, availability of the tool in the laboratory or its ease of use. This poses a great challenge when providing reproducible results and creates uncertainty regarding the reliability of the information derived. This is a major barrier to the implementation of SMg approaches in routine clinical microbiology laboratories.

In this study, the aim was to identify the critical steps when using SMg for the identification and characterization of microbial pathogens directly from clinical specimens using methods that are likely to be available in clinical microbiology laboratories wanting to implement genomics for pathogen identification or molecular epidemiology studies. For this purpose, we used three human-DNA depletion kits and evaluated a diverse set of bioinformatics tools (commercial and non-commercial) in order to investigate how well they performed and what the differences would be in terms of taxonomic classification, antimicrobial resistance gene detection and typing directly from patient samples, bypassing culture.

## Results

### Classical identification

Nine body fluid samples and one tissue sample from 9 different patients were sequenced, including one sample from peritoneal fluid, five from pus (3 abscesses and 2 empyemas), two from synovial fluid of knees with prosthesis, one from sputum and one from a bone biopsy (Table 1). In total 15 different isolates obtained from the 10 samples were considered of possible clinical significance and were selected for species identification and antimicrobial susceptibility testing during routine work up of the samples (Tables 2, 3 and 4). In samples 2 and 3, only one colony-forming unit (CFU) of *Escherichia coli* and *Staphylococcus epidermidis*, respectively, was detected after 48 hours of incubation. In samples 2 and 5, the anaerobic cultures were mixed to such an extent, that no further characterization of the colonies was performed, and the results were reported as anaerobic mixed culture.

**Table 1:** Characteristics of the samples and mapping of trimmed reads against a human genome hg19 (%) using CLC Genomics Workbench v10.0.1.

	Sample 1	Sample 2	Sample 3	Sample 4	Sample 5	Sample 6	Sample 7	Sample 8	Sample 9	Sample 10	Negative control
Sample type	Peritoneal fluid	Pus (abscess)	Synovial fluid	Synovial fluid	Pus (abscess)	Pus (empyema)	Pus (empyema)	Bone biopsy	Pus (abscess)	Sputum	Water
DNA extraction method	Ultra-Deep Microbiome Prep (Molzym)	Ultra-Deep Microbiome Prep (Molzym)	Ultra-Deep Microbiome Prep (Molzym)	Ultra-Deep Microbiome Prep (Molzym)	Ultra-Deep Microbiome Prep (Molzym)	QIAamp DNA Microbiome Kit (Qiagen)	QIAamp DNA Microbiome Kit (Qiagen)	Micro-DX^TM^ (Molzym)	Micro-DX^TM^ (Molzym)	Micro-DX^TM^ (Molzym)	QIAamp DNA Microbiome Kit (Qiagen)
Total number of reads	5,892,978	9,603,346	8,615,810	6,078,166	8,368,930	2,912,802	1,486,700	6,534,866	6,173,132	7,596,836	1,730,738
Mapped reads against hg19	5,249,063 (89.2%)	7,828.746 (81.6%)	8,254,594 (95.9%)	6,015,945 (99.0%)	309,588 (3.7%)	2,877,066 (98.8%)	922,932 (62.2%)	229,149 (3.5%)	6,081,612 (98.5%)	7,337,832 (96.7%)	1,706,861 (98.9%)
Unmapped reads	632,951 (10.8%)	1,770,558 (18.4%)	355,200 (4.1%)	61,099 (1.0%)	8,052,272 (96.3%)	34,506 (1.1%)	561,772 (37.8%)	6,303,803 (96.5%)	89,922 (1.5%)	235,520 (3.3%)	19,805 (1.2%)

**Table 2:** Microorganisms identified by conventional methods, WGS and using shotgun metagenomics and the taxonomic classification methods in Unix.

Sample number	Culture result (CFU)^a^	Conventional identification (MALDI-TOF)	WGS-based identification	Shotgun metagenomics		
				Kraken^b^	MIDAS^c^	MetaPhlAn^c^
1	10^3^ 10^3^ 10	*E. faecium* *S. haemolyticus* *C. glabrata*	*E. faecium* *S. haemolyticus* —	*E. faecium* (34.6%) *S. haemolyticus* (10.1%) —	*E. faecium* (62.0%) *S. haemolyticus* (28.0%) —	*E. faecium* (66.6%) *S. haemolyticys* (27.7%) —
2	10^3^ 1 Not determined	*E. avium* *E. coli* Anaerobes	—^#^ —^#^ —^#^	Not identified* Not identified* Several species (29.5%)	Not identified* Not identified* Several species (100.0%)	Not identified* Not identified* Several species (100.0%)
3	1	*S. epidermidis*	—^#^	*S. aureus* (0.2%)	Not identified*	Not identified*
4	10^3^	*S. aureus*	*S. aureus*	*S. aureus* (0.73%)	*S. aureus* (100%)	*S. aureus* (100%)
5	≥10^5^ ≥ 10^5^ 10^3^ 10^3^ Not determined 10	*E. coli* *K. oxytoca* *S. anginosus* *E. faecalis* Anaerobes *C. albicans*	*E. coli* *K. oxytoca* —^#^ *E. faecalis* —^#^ —^#^	*E. coli* (9.7%) *K. oxytoca* (0.5%) *S. anginosus* (0.07%) *E. faecalis* (0.3%) Several species (12.7%) —	*E. coli* (6.5%) *K. oxytoca* (0.3%) *S. anginosus* (0.01%) *E. faecalis* (0.9%) Several species (96.7%) —	*E. coli* (8.5%) *K. oxytoca* (0.3%) *Streptococcus* spp. (0.09%) *E. faecalis* (0.7%) Several species (90.4%) —
6	10^3^	*E. faecium*	*E. faecium*	*E. faecium* (0.77%)	Not identified*	Not identified*
7	10^2^	*S. aureus*	—^#^	*S. aureus* (82.9%)	*S. aureus* (100%)	*S. aureus* (100%)
8	10^3^	*O. intermedium*	*O. intermedium*	*O. anthropi* (21.3%)	*O. intermedium* (99.4%)	*O. intermedium* (99.1%)
9	10^3^	*S. aureus*	*S. aureus*	*S. aureus* (22.9%)	*S. aureus* (100%)	*S. aureus* (100%)
10	10^3^	*S. marcescens*	—^#^	*S. marcescens* (64.7%)	*S. marcescens* (99.1%)	*S. marcescens* (100%)

^a^The number of colonies of a given species was estimated from the number of colonies with the same morphology on the same plate; ^b^The relative abundance is calculated using total number of reads as denominator; ^c^The relative abundance is calculated with the total number of classified reads as denominator; ^d^miniKraken database was used; ^#^Although there was a laboratory identification, no isolates were available for WGS; *No reads matched that specific pathogen, not even at the genus level.

**Table 3:** Microorganisms identified by conventional methods, WGS and using shotgun metagenomics and the taxonomic classification methods in CLC Genomics Workbench.

Sample number	Culture result (CFU)^a^	Conventional identification (MALDI-TOF)	WGS-based identification	Shotgun metagenomics	
				Taxonomic Profiling (CLC)^b^	Best match with K-mer spectra (CLC)^c^
1	10^3^ 10^3^ 10	*E. faecium* *S. haemolyticus* *C. glabrata*	*E. faecium* *S. haemolyticus* —	*E. faecium* (71%) *S. haemolyticus* (24%) *C. glabrata* (100%)	*E. faecium* (41.4%) *S. haemolyticus* (13.8%) *C. glabrata* (0.5%)
2	10^3^ 1 Not determined	*E. avium* *E. coli* Anaerobes	—^#^ —^#^ —^#^	Not identified* Not identified* Several species (97%)	Not identified* Not identified* Several species (13.2%)
3	1	*S. epidermidis*	—^#^	Not identified*	*S. aureus* (4%)
4	10^3^	*S. aureus*	*S. aureus*	Not identified*	*S. aureus* (9.7%)
5	≥10^5^ ≥ 10^5^ 10^3^ 10^3^ Not determined 10	*E. coli* *K. oxytoca* *S. anginosus* *E. faecalis* Anaerobes *C. albicans*	*E. coli* *K. oxytoca* —^#^ *E. faecalis* —^#^ —^#^	*E. coli* (25%) *K. michiganensis* (0.3%) Not identified* E. faecalis (2%) Several species (70.0%) Not identified*	*E. coli* (11.5%) Not identified* Not identified* E. faecalis (0.6%) Not identified* C. albicans (<0.05%)
6	10^3^	*E. faecium*	*E. faecium*	Not identified*	*E. faecium* (4.0%)
7	10^2^	*S. aureus*	—^#^	*S. aureus* (100%)	*S. aureus* (95.5%)
8	10^3^	*O. intermedium*	*O. intermedium*	*O. intermedium* (86.0%)	*O. intermedium* (91.2%)
9	10^3^	*S. aureus*	*S. aureus*	*S. aureus* (100%)	*S. aureus* (81.2%)
10	10^3^	*S. marcescens*	—^#^	*S. marscescens* (100%)	*S. marcescens* (79.7%)

^a^The number of colonies of a given species was estimated from the number of colonies with the same morphology on the same plate; ^b^The relative abundance is calculated with the total number of classified reads as denominator; ^c^Based on the Output Quality Report; ^#^Although there was a laboratory identification, no isolates were available for WGS; *No reads matched that specific pathogen, not even at the genus level.

**Table 4:** Microorganisms identified by conventional methods, WGS and using shotgun metagenomics and the taxonomic classification methods in webpages (BaseSpace, Taxonomer and CosmosID).

Sample number	Culture result (CFU)^a^	Conventional identification (MALDI-TOF)	WGS-based identification	Shotgun metagenomics				
				Genius (Basespace)^c^	Kraken (Basespace)^c,d^	MetaPhlAn (Basespace)^c^	Taxonomer (Utah)^b,e^	Cosmos ID^a^
1	10^3^ 10^3^ 10	*E. faecium* *S. haemolyticus* *C. glabrata*	*E. faecium* *S. haemolyticus* —	*E. faecium* (14.4%) *S. haemolyticus* (55.8%) —	*E. faecium* (25.0%) *S. haemolyticus* (20.1%) —	*E. faecium* (65.1%) *S. haemolyticys* (30.4%) —	*E. faecium* (22.9%) *S. haemolyticus* (20.1%) Not identified*	*E. faecium* (50.3%) *S. haemolyticus* (22.1%) *C. glabrata* (88.6%)
2	10^3^ 1 Not determined	*E. avium* *E. coli* Anaerobes	—^#^ —^#^ —^#^	Not identified* Not identified* Several species (94.0%)	Not identified* Not identified* Several species (27.0%)	Not identified* Not identified* Several species (54.2%)	Not identified* Not identified* Several species (14.2%)	Not identified* Not identified* Several species (100%)
3	1	*S. epidermidis*	—^#^	*S. aureus* (100%)	*S. aureus* (0.1%)	Not identified*	*S. pseudintermedius* (3.4%)	Not identified*
4	10^3^	*S. aureus*	*S. aureus*	*S. aureus* (100%)	*S. aureus* (0.3%)	*S. aureus* (100%)	*S. aureus* (8.3%)	*S. aureus* (100%)
5	≥10^5^ ≥ 10^5^ 10^3^ 10^3^ Not determined 10	*E. coli* *K. oxytoca* *S. anginosus* *E. faecalis* Anaerobes *C. albicans*	*E. coli* *K. oxytoca* —^#^ *E. faecalis* —^#^ —^#^	*E. coli* (0.4%) Not identified* S. anginosus (0.03%) *E. faecalis* (0.8%) Several species (45.0%) —	*E. coli* (10.2%) *K. oxytoca* (0.5%) *S. anginosus* (0.4%) *E. faecalis* (0.3%) Several species (8.0%) —	*E. coli* (7.0%) *K. pneumoniae* (0.01%) *S. anginosus* (0.3%) *E. faecalis* (0.7%) Several species (89.1%) —	*E. coli* (3.6%) *K. michiganensis* (0.1%) *S. anginosus* (0.1%) *E. faecalis* (0.1%) Several species (60.3%) —	*E. coli* (7.6%) *K. oxytoca* (1.7%) *S. anginosus* (0.09%) *E. faecalis* (3.7%) Several species (86.2%) Not identified*
6	10^3^	*E. faecium*	*E. faecium*	*E. faecium* (4.2%)	*E. faecium* (14.8%)	*E. faecium* (5.5%)	*E. faecium* (1.4%)	*E. faecium* (4.1%)
7	10^2^	*S. aureus*	—^#^	*S. aureus* (100%)	*S. aureus* (93.8%)	*S. aureus* (100%)	*S. aureus* (14.2%)	*S. aureus* (100%)
8	10^3^	*O. intermedium*	*O. intermedium*	*O. intermedium* (100%)	*O. nthropic* (88.9%)	*O. intermedium* (99.8%)	*O. intermedium* (13.1%)	*O. intermedium* (49.5%)
9	10^3^	*S. aureus*	*S. aureus*	*S. aureus* (100%)	*S. aureus* (99.5%)	*S. aureus* (100%)	*S. aureus* (12.7%)	*S. aureus* (100%)
10	10^3^	*S. marcescens*	—^#^	*S. marcescens* (32.5%)	*S. marcescens* (94.8%)	*Serratia* spp. (100%)	*S. marcescens* (1.4%)	*S. marscescens* (38.4%)

^a^The number of colonies of a given species was estimated from the number of colonies with the same morphology on the same plate; ^b^The relative abundance is calculated using total number of reads as denominator; ^c^The relative abundance is calculated with the total number of classified reads as denominator; ^d^miniKraken database was used; ^e^Full Analysis mode was used; ^#^Although there was a laboratory identification, no isolates were available for WGS; *No reads matched that specific pathogen, not even at the genus level.

Antimicrobial susceptibility testing, revealed three isolates to be fully susceptible, while the others were resistant to at least one antimicrobial. Two isolates, one *Staphylococcus haemolyticus* and one *S. epidermidis* were oxacillin-resistant and positive in the cefoxitin test (Vitek 2).

There was fungal growth in 2 samples (1 and 5) that included two *Candida* species (one *Candida glabrata* and one *Candida albicans*). The different bacterial and fungal species identified in each sample are shown in Tables 2, 3 and 4.

### Comparison of standard procedures and shotgun metagenomics for the identification of clinically relevant pathogens

**Figure 1:**
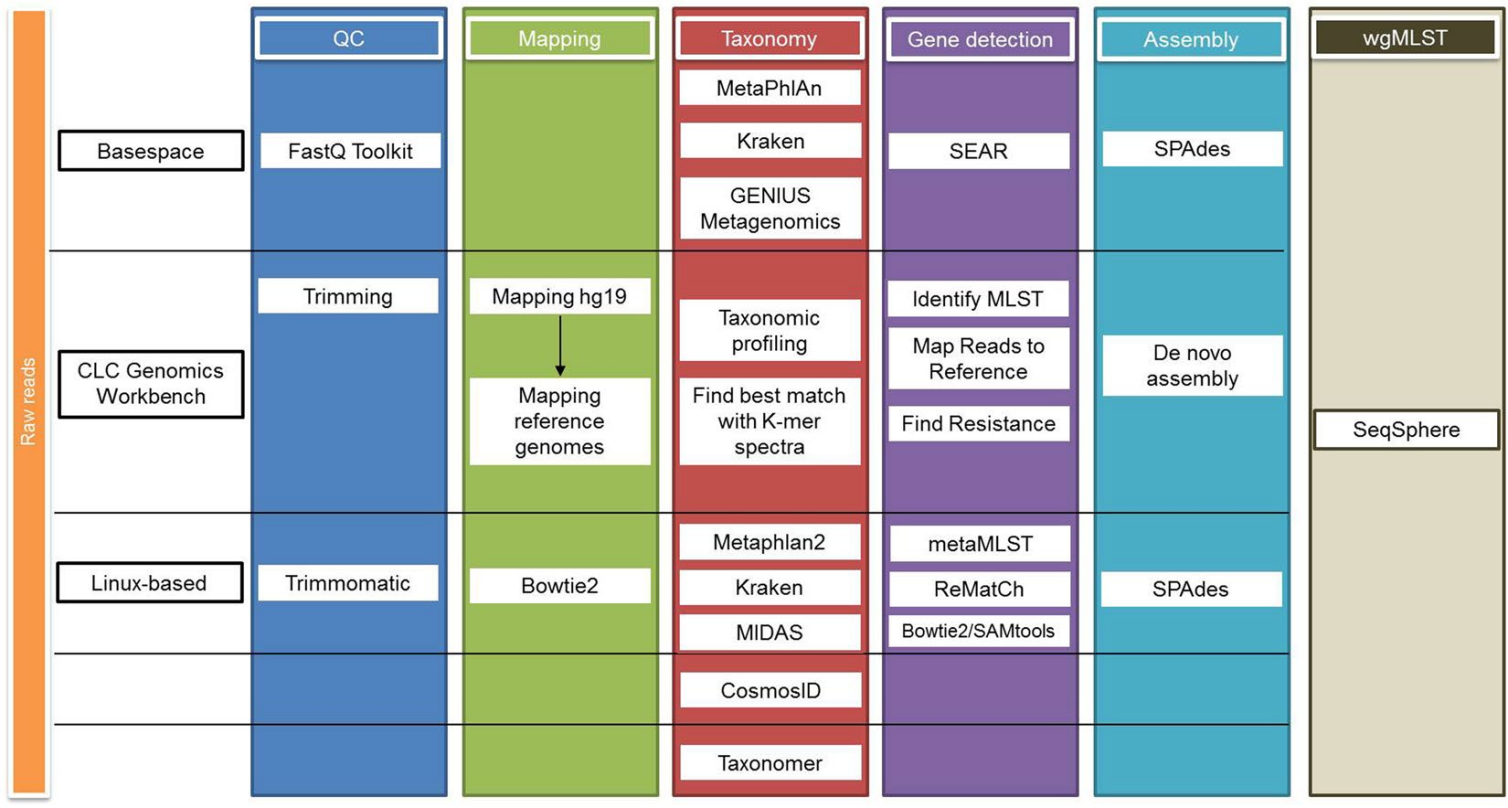
Scheme of the bioinformatic analysis of the metagenomics samples.

The tools used for taxonomic classification are shown in Fig. 1. The total number of reads and the total number of reads mapped against the human genome (hg19) varied between samples, ranging from 3.5% to 98.9% (Table 1). The abundance of human reads was not determined by the type of sample but was probably influenced by individual characteristics of each sample and the success of the methods used in depleting the human DNA. We identified the microorganisms present using different taxonomical methods, including three Unix-based tools (Kraken, Metaphlan2 and MIDAS), web-based tools including both commercial and freely available solutions (BaseSpace, Taxonomer and CosmosID) and one commercial approach having a graphical interface (CLC Genomics Workbench v10.0.1). The taxonomic classification results for each sample are presented in Tables 2, 3 and 4. In 8 samples, all the microorganisms identified by classical culture were also identified through metagenomics (using at least one method). In sample 2, two of the bacterial species identified by classical culture, i.e., *E. coli* and one *Enterococcus avium* were not identified through shotgun metagenomics and in sample 3 there was no concordance between the results of MALDI-TOF and the taxonomical classification methods at the species level (Tables 2, 3 and 4). We identified *Ochrobactrum intermedium* in the negative control, but in low amounts (1.0% of the reads mapped to the reference genome with the accession number NZ_ACQA01000002 and only 1.4% of the reference genome was covered). The sensitivity and positive predictive value of each classification method is shown in Table 5.

**Table 5:** Performance of the different taxonomic classification methods for each sample. Sensitivity and positive predictive value were calculated using culture/MALDI-TOF as standards.

Method	Total number of bacteria identified^a^	True positives^a^	False positives	False negatives	Sensitivity (%)	PPV (%)
Culture/MALDI-TOF	9	9	0	0	100%	100%
MetaPhlAn (BaseSpace)	16	7	9	2	78%	44%
Genius (BaseSpace)	35	8	27	1	89%	23%
Kraken (BaseSpace)	959	7	952	2	78%	1%
Taxonomer (Full Analysis)	4649	8	4641	1	89%	0%
CosmosID	35	8	27	1	89%	23%
Taxonomic Profiling (CLC Genomics Workbench v10.0.1)	17	6	11	3	67%	35%
Best match K-mer spectra (CLC Genomics Workbench v10.0.1)	12	8	4	1	89%	67%
Kraken (Unix)	198	7	191	2	78%	4%
MetaPhlAn2 (Unix)	15	7	6	4	75%	75%
MIDAS (Unix)	34	7	26	2	88%	50%

^a^Excluding the samples with non-identified anaerobic bacteria (Samples 2 and 5).

Abbreviations: PPV, positive predictive value.

### Determination of antimicrobial resistance

Metagenomics provides other sequence information in addition to pathogen detection. We determined the presence of antimicrobial-resistance genes in the SMg sequence data and compared the results with those obtained from WGS and phenotypic resistance testing (Table 6).

**Table 6:** Antimicrobial resistance phenotypes and antimicrobial resistance genes detected using different approaches.

Sample number	Conventional identification (MALDI-TOF)	Conventional susceptibility testing (VITEK 2)^b^	WGS CLC Genomics Workbench	Shotgun metagenomics	
				ReMatCh (Unix)	CLC Genomics Workbench^a^
1	*E. faecium* *S. haemolyticus*	LEV, ERY, CLI OXA, GEN, CIP, FOS, ERY, CLI	*erm(B)*, *msr(C), ant(6’)-Ia, aph(3’)-III, dfrG* *blaZ, mecA, ant(6’)-Ia, aph(3’)-III, aac(6’)-aph(2”), erm(C), mph(C), msr(A), dfrG*	*erm(B)*, *msr(C), ant(6’)-Ia, aph(3’)-III, aac(6’)-aph(2”), blaZ, mecA, erm(C), mph(C), msr(A), dfrG*	*erm(B)*, *msr(C), ant(6’)-Ia, aph(3’)-III, aac(6’)-aph(2”), blaZ, mecA, erm(C), mph(C), msr(A), dfrG*
2	*E. avium* *E. coli* Anaerobes	DOX, CLI susceptible —	—^#^ —^#^ —^#^	Not detected Not detected *catS, lnu(D), lsa(C), cepA-44, tet(Q)*	Not detected Not detected *catS, lnu(D), lsa(C), cepA-44, tet(Q), fusA*
3	*S. epidermidis*	OXA, GEN, TEC, FUS, CIP, ERY, CLI	—^#^	Not detected	Not detected
4	*S. aureus*	PEN, ERY	*blaZ, spc, erm(A)*	Not detected	Not detected
5	*E. coli* *K. oxytoca* *S. anginosus* *E. faecalis* Anaerobes	susceptible AMX susceptible DOX, CLI —	—^#^ *blaOXY-1–3* —^#^ *tet(M), lsa(A)* —^#^	— Not detected — *tet(M)* *cfxA4, tet(Q)*	— Not detected — *tet(O)* *cfxA4, tet(Q)*
6	*E. faecium*	PEN, AMX, CFX, IMP, GENhl, STRhl, LEV, ERY, CLI, AMP/SUL	*erm(B)*, *msr(C), ant(6’)-Ia, aph(3’)-III, aac(6’)-aph(2”), dfrG*	Not detected	Not detected
7	*S. aureus*	PEN	*blaZ*	*blaZ, norA*	*blaZ*
8	*O. intermedium*	AMX, PIP/TAZ, CFX, CFT, CTZ, IMP, FOX, TOB, FOS, NIT, TMP	*bla*OCH-2	*bla*OCH-5	*bla*OCH-2
9	*S. aureus*	PEN	—^#^	*blaZ*	*blaZ*
10	*S. marcescens*	AMX, AMC, CFX, FOX, NIT, POL	—^#^	*blaSST-1, tet(41), oqxB, aac(6’)-Ic*	*tet(41), oqxB, aac(6’)-Ic*

^a^The analysis aborted when the script tried to connect to NCBI.

^b^Only non-susceptibility is indicated. Abbreviations: AMP/SUL, ampicillin/sulbactam; AMX, amoxicillin; AMC, amoxicillin/clavulanate; CFX, cefuroxime; FOS, fosfomycin; FOX, cefoxitin; CIP, ciprofloxacin; CLI, clindamycin; DOX, doxycycline; ERY, erythromycin; FUS, fusidic acid; GEN, gentamicin; GENhl, gentamicin high-level; LEV, levofloxacin; NIT, nitrofurantoin; PEN, penicillin; POL, polymyxin B; STRhl, streptomycin high-level; TEC, teicoplanin.

Antimicrobial resistance genes found with CLC Genomics Workbench and ReMatCh in samples 1, 7 and 9 correlated well with phenotypic results. However, in the other 7 samples, not all antimicrobial resistance genes that could explain the phenotypic profile were identified. In addition, in samples 2, 5, 7 and 10, ReMatCh detected different resistance genes compared to those reported by CLC Genomics Workbench (Table 6). Some of these differences (genes *norA*, *bla*SST-1, *fusA*) were due to slight differences in the databases used, however, the other resistance genes were present in both databases. Interestingly, in two samples (samples 2 and 5), we were able to identify several antimicrobial resistance genes usually found in anaerobic bacteria. These were not reported by classical microbiology methods, probably because they were not considered relevant pathogens worthy of subsequent susceptibility study (mixed anaerobic culture).

The SEAR app in BaseSpace (the only one available for antimicrobial resistance gene detection) crashed several times, although we performed the analysis repeatedly, using different parameters. We were only able to get results in 3 samples, with no resistance genes detected.

### MLST and wgMLST analysis

**Figure 2:**
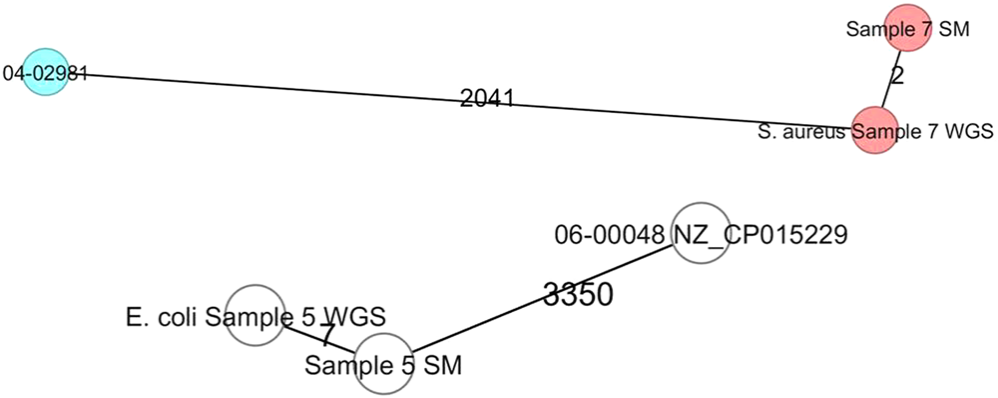
Minimum-spanning tree based on wgMLST allelic profiles of two S. aureus genomes and two *E. coli* genomes obtained through SM and WGS in comparison to reference strains 04-02981 (GenBank accession number NC_017340) and 06-00048 (NZ_CP015229), respectively. Each circle represents an allelic profile based on sequence analysis. The numbers on the connecting lines illustrate the numbers of target genes with differing alleles.

In three cases when SMg data covered ≥ 93% of the genome we were able to identify the ST, which corresponded to the one found using WGS of the isolated bacteria using CLC Genomics Workbench (n = 2) and metaMLST (n = 1). These results are summarized in Table 7. Assembled genomes and metagenomes, were compared by wgMLST analysis using Ridom SeqSphere+. Figure 2 shows examples of the allele difference between the genomes obtained through WGS versus the genomes obtained through shotgun metagenomics.

**Table 7:** Results of MLST using by whole genome sequencing and shotgun metagenomics.

Sample number	Conventional identification (MALDI-TOF)	WGS	Shotgun metagenomics	
		CLC Genomics Workbench v10.1.1	CLC Genomics Workbench v10.1.1	metaMLST (Unix-based)
1	*E. faecium* *S. haemolyticus*	ST117 ST25	Not detected (6 alleles identified correctly) Not detected (3 alleles identified correctly)	ST117 Not detected
2	*E. avium* *E. coli* Anaerobes	—^#^ —^#^ —^#^	— Not detected —	— Not detected —
3	*S. epidermidis*	—^#^	Not detected	Not detected
4	*S. aureus*	ST30	Not detected	Not detected
5	*E. coli* *K. oxytoca* *S. anginosus* *E. faecalis* Anaerobes	ST141 ST40 —^#^ ST179 —^#^	ST141 Not detected — Not detected —	ST4508 Not detected — Not detected —^#^
6	*E. faecium*	ST117	Not detected	Not detected
7	*S. aureus*	ST30	ST30	ST667
8	*O. intermedium*	—	—	—
9	*S. aureus*	—^#^	Not detected	Not detected
10	*S. marcescens*	—^#^	—	—

Abbreviations: ST, sequence type.

### Characterization of mobile genetic elements

**Figure 3:**
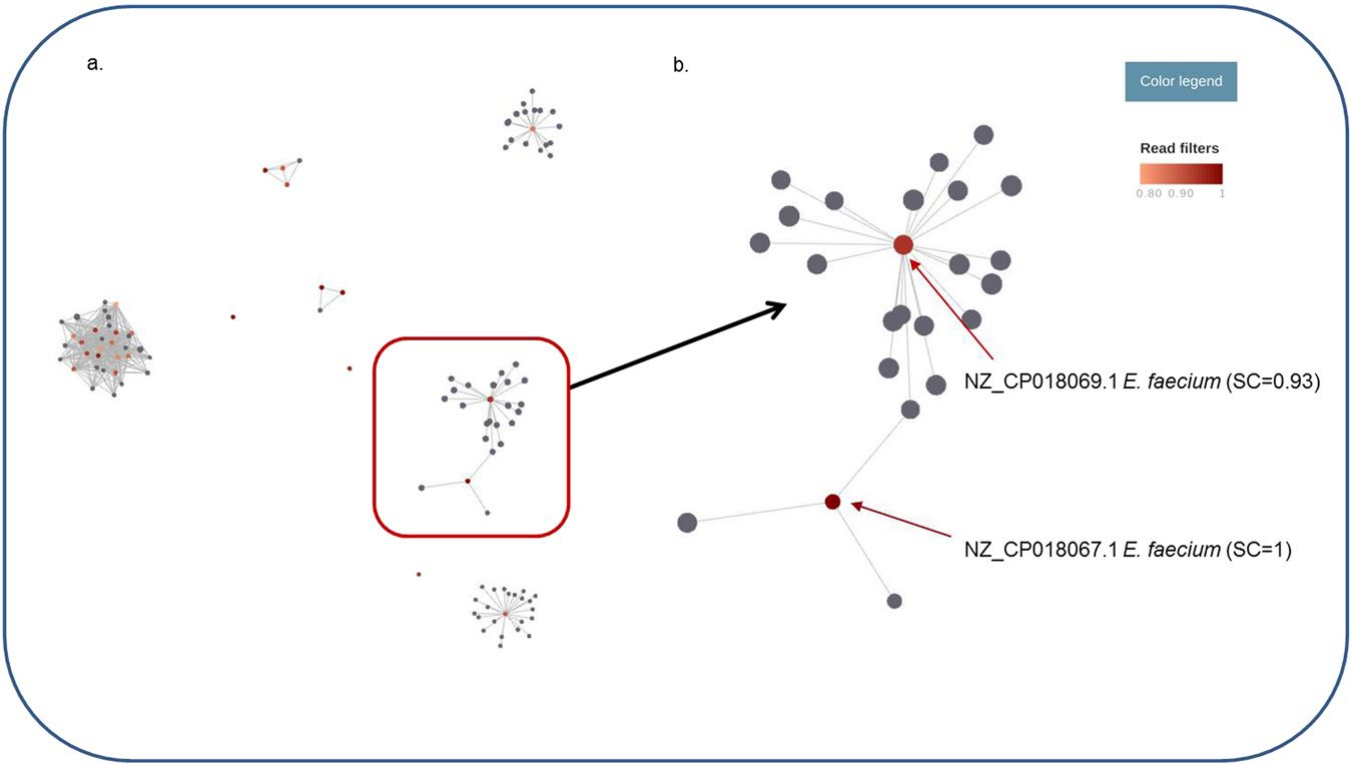
(**a**) Overview of the nodes (representing plasmid sequences) and links between plasmids (connecting similar plasmids) found in Sample 1 (SMg) using the pATLAS tool. (**b**) A closer look at one of the cloud of plasmids. The color gradient in each cloud of plasmids represents the plasmid sequence coverage (SC), varying between 0–0.79 (grey) and 0.80–1 (red gradient).

**Figure 4:**
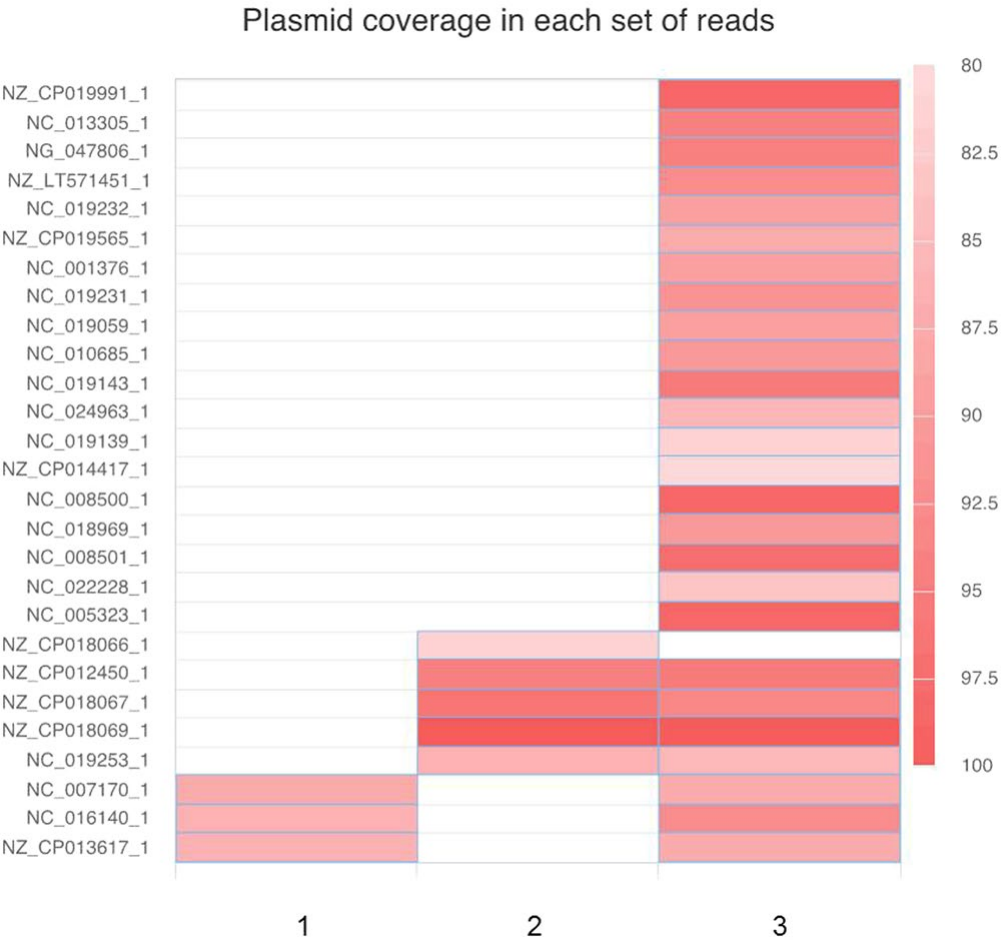
A heatmap comparing the identified plasmids using bowtie2 in *S. haemolyticus* WGS (1), *E. faecium* WGS (2) and in the SMg dataset (3) isolated from sample 1.

Two different approaches, i.e. CLC Genomics Workbench and Bowtie2 were used to identify plasmids present in the sequence data. Both approaches used mapping of sequences against the same plasmid database. Since some plasmids present in the database are very similar and sequence reads may be mapped to more than one plasmid, we used the pATLAS tool, which provides an overview of the nodes (representing plasmid sequences) and links between plasmids (which connect similar plasmids), to enable the visualization of the plasmids identified (Fig. 3). A color gradient indicates the sequence coverage of the plasmids. In most cases, the same plasmids were identified by both approaches, with some small differences in sequence coverage. When comparing the plasmids identified in the SMg dataset versus the WGS data, most of the plasmids were also detected in the isolates (an example is shown in Fig. 4). However, some plasmids were not identified in any of the isolated bacteria and were probably residing in low-abundant species.

## Discussion

This study evaluated the suitability of SMg for the microbiological diagnosis and (patho- and epi-) typing of microorganisms directly from real patient samples. The whole procedure took between 48–54 hours to complete, which is shorter than culture-based methods if one includes typing. However, the amount of information derived from SMg in most cases, did not overcome the necessity for pathogen isolation and subsequent (phenotypic and genotypic) typing, which can take up to 1–2 weeks (particularly in slow-growing organisms). Nevertheless, SMg can help guide antimicrobial therapy and can be helpful in cases where there is a suspicion of transmission and there is a need to quickly determine the genetic relationship between pathogens, although the success of SMg in individual patient samples can be highly variable, as reported here.

Different bioinformatics pipelines were evaluated to identify potential differences between them and identify those which could provide the clinical microbiologist with the maximum of relevant and accurate information. In terms of microbial identification, in both Unix and web-based approaches we would recommend MetaPhlAn, since it has good sensitivity and a good positive predictive value (PPV). The Find Best Match K-mer Spectra tool should be used in the context of the CLC Genomics Workbench, since it had a higher sensitivity and PPV compared to the Taxonomic Profiling tool.

In a clinical setting, a combination of high sensitivity and high PPV of any new method is key. Popular software designed for bacterial identification can predict dozens to hundreds of species in *in vitro* generated bacterial communities of known composition^8^. We observed the same when using Kraken and Taxonomer when comparing to culture-based methods. For both Kraken and Taxonomer, relative abundance cut-off values may be required to limit the number of species identified. However, which cut-off values should be used are a matter of debate, since in some cases, even if applying a cut-off value as low as 1.0% (comparable to what was found in the negative control) would have resulted in decreased sensitivity (e.g. the *Streptococcus anginosus* identified by culture in Sample 5 would have been disregarded). The methods that employ several parameters to infer microbial identification are superior, because they not only rely on the relative abundance of bacterial species, but also on the genome coverage and on the proportion of the genome that was covered. On the other hand, in some cases SMg may be more sensitive than culture in identifying pathogens, reflecting the higher sensitivity or the capacity to detect bacterial species which are non-culturable in the conditions used or that are no longer culturable, such as due to prior antimicrobial therapy. In such cases, other methods like 16 S rRNA gene sequencing or the recently described 16S-23S rRNA-coding region sequencing method^9^ may be used for discrepancy analyses. However, here we decided to use culture-based methods as the gold standard, since this is still the method of choice in clinical microbiology.

One limitation of this study was the exclusion of culture-negative samples and thus their inclusion would have affected the calculation of the specificity values. However, as mentioned above, culture-negative samples do not necessarily mean that the samples are pathogen-free, but it might only reflect the low sensitivity or capacity of culture-based methods to detect non-culturable bacterial species. As with other (molecular) methods, several controls should be included to validate the obtained results, including a negative control. In our negative control, we detected an *O. intermedium* strain, although with only 1.0% of the reads mapping to the reference genome and covering only 1.4% of the reference genome (accession number NZ_ACQA01000002). These results may be due to contamination during library preparation (e.g. sample-to-sample contamination prior to indexing), the result of sequencing artefacts (e.g. demultiplexing errors), or to incorrect classification during data analysis (e.g. highly similar regions)^3^. Our samples and sequencing libraries were handled in laminar flow cabinets; however, we cannot also exclude the possibility of contamination. Furthermore, the reagents used may also be or become contaminated with DNA leading the detection of these contaminating species, something that has been described previously^7^. This poses a challenge for interpretation, because some positive samples also had very low numbers of reads for some pathogens (<1%). When approaching this limit of detection, small numbers of pathogen reads will be difficult to interpret, as they can represent true-positives with low abundance in the sample, or artifacts such as contamination during library preparation^3^.

In terms of antimicrobial resistance gene detection, ReMatCh (Unix) and the CLC Genomics Workbench Find Resistance tool gave comparable results. Since ReMatCh (Unix) performs the analysis at the read level, while CLC Genomics Workbench performs it at the contig level, we suggest that both strategies should be employed in parallel when looking for antimicrobial resistance genes. It is also important to emphasize that the contig-level approach employed by CLC Genomics Workbench may give negative results if the sequence coverage is set to a high percentage (e.g. above 80%). This is due to the assembly method, which may split the antimicrobial resistance genes into different contigs, when the number of reads is too low. This phenomenon was observed in Sample 1, for the *aac(6’)-aph(2”)* gene, which was split into three different contigs, each part corresponding to less than 40% of the gene. Only when applying a cut-off value of ≥20% for sequence coverage could we identify all three parts of the gene, which in total corresponded to 89% of the entire sequence. Finally, it is important to point out that the ResFinder database (used here), and other databases, focus on acquired genes, not including chromosomal point mutations resulting in antimicrobial resistance. However, a recently developed tool, PointFinder, was added to ResFinder for the detection of chromosomal point mutations associated with antimicrobial resistance^10^ and an updated database will be available soon.

Another challenge is to infer where these antimicrobial resistance genes are located (chromosome or plasmid). The study of mobile genetic elements, including plasmids, carrying antimicrobial resistance genes present in clinical samples is important to predict possible treatment failures and the spread of resistance within and across bacterial species. When performing bacterial isolation followed by WGS, information on polymicrobial infections may be lost. This is mainly driven by a bottleneck in culture, where some bacterial species are not isolated with standard work up protocols (frequently anaerobes and slow-growing organisms). The presence of antimicrobial resistance genes in plasmids of bacteria other than those isolated through culture poses a risk since they are not identified by conventional methods but could potentially be horizontally transmitted to pathogenic bacteria under the antimicrobial selective pressure of treatment. Antimicrobial administration may also select minority populations where these resistance determinants are found. Furthermore, the understanding of how plasmids are shared by different bacteria in a bacterial community (e.g. within an infection site or in the gut) can improve our understanding of how these elements disseminate across species and from patient to patient^11^. The SMg approach is clearly more efficient than culture in identifying the “cloud” of plasmids present in a given sample (Fig. 4) and which can be potentially transferred to more pathogenic species generating problems of resistance, as was the case with the emerge of vancomycin resistance *S. aureus*^12^.

Whole-genome sequencing has been used extensively for several purposes^13^ and is considered to have the potential of playing an important role in clinical microbiology^14^. It is the ongoing goal of medical molecular microbiology to develop faster typing methods that can be used for outbreak surveillance. For this purpose, we assembled the metagenomics data and compared it with the assemblies given by WGS. Surprisingly, the assemblies provided by SPAdes in BaseSpace were closer to the assemblies provided by WGS. When comparing the genomes obtained through WGS and SMg, we could see that in 4 out of 8 bacterial isolates the number of different alleles was ≤7. This showed the potential of SMg to draw phylogenetic relationships from uncultured bacterial genomes, although more potentially limited than those obtained using WGS data from axenic cultures. As for the detection of resistance genes, a key limiting factor may be the number of bacterial reads, reflected in a lower genome coverage (e.g. samples 4 and 6). In these cases, we would have to either improve the human-DNA depletion step, improve the microbial enrichment or perform sequencing at a higher sequencing depth to have enough microbial reads to be able to get a more appropriate genome coverage. Yet, this last step will severely raise the sequencing costs, which might render the methodology unfeasible for routine application.

In this study, we evaluated the results of metagenomics pipelines using three different methods. CLC Genomics Workbench has advantages over the other methods. It does not require previous knowledge of Unix-based tools, it is arguably the most user-friendly and delivered reliable results for microbial identification and antimicrobial resistance gene detection. The downside was the assembly approaches, which provided lower wgMLST allele detection, when compared to the assemblies using SPAdes (BaseSpace and Unix). BaseSpace, the other commercial solution, on the other hand, provided only a few tools that can be used for metagenomics data. Furthermore, since Illumina did not develop the apps themselves, they offered no direct support. Contacting the developers (via email and posting on their forum) does not guarantee a solution to the issues in a time frame compatible with a routine clinical microbiology laboratory work. The dependence and no direct control over a third party to resolve software bugs and provide a stable platform illustrates a disadvantage of a cloud-based system like BaseSpace. Finally, the Unix-based pipeline complemented the data on antimicrobial resistance genes but did not offer better results in terms of microbial identification and MLST typing. However, many more freely available tools for this last purpose could have been used, potentially improving on the results obtained. Reference-guided assembly approaches, taking advantage of the species information derived in the first steps of our analysis pipelines, will deserve further study in the future since these may provide higher quality assemblies from metagenomics data. The main advantage of an open-source approach is its flexibility since it allows the user to choose the most adequate method for each desired outcome.

There were several limitations to this study. First, the number of samples included was low and some of the bacterial isolates were not available for further WGS analysis. However, the extended data analyses performed in each sample limited the number of samples to be included. It is our intention to move forward with the most adequate pipelines for each purpose and apply them to additional patients’ samples. Second, the samples differed greatly from each other. However, in our point of view, this was beneficial to the study, since it did not bias the analyses as it could have happened if only one type of sample had been used. Finally, we used three different extraction methods that could have influenced the final results. Yet, as can be seen in Table 1, the number of human reads differed between samples, even when using the same extraction kit. This suggests none of the kits is clearly superior to the others and that the ratio between host and microbial DNA or other individual sample characteristics will be the major determinants of the proportion of microbial reads.

In conclusion, this study showed the potential but also highlighted the problems of implementing shotgun metagenomics for the identification and typing of pathogens directly from clinical samples. Based on the results obtained here we can conclude that the tools and databases used for taxonomic classification and antimicrobial resistance will have a key impact on the results, cautioning about the comparison between studies using different methods and suggesting that efforts need to be directed towards standardization of the analysis methods if SMg is to be used routinely in clinical microbiology.

## Methods

### Sample collection

Nine body fluid samples and one tissue sample entering the Medical Microbiology laboratory were selected for metagenomics sequencing. These included one sample from peritoneal fluid, five from pus (3 abscesses and 2 empyema), two from synovial fluid of knees with prosthesis, one from sputum and one from a bone biopsy (Table 1). All samples were stored at 4 °C for a variable period (2–10 days). The samples used for the present analyses were collected during routine diagnostics and infection prevention and control investigations. All procedures were carried out according to guidelines and regulations of UMCG concerning the use of patient materials for the validation of clinical methods, which are in compliance with the guidelines of the Federation of Dutch Medical Scientific Societies (FDMSS). Every patient entering the UMCG is informed that samples taken may be used for research and publication purposes, unless they indicate that they do not agree to it. This procedure has been approved by the Medical Ethical Committee of the UMCG. Informed consent was obtained from all individuals or their guardians prior to study participation. All samples were used after performing and completing a conventional microbiological diagnosis and were coded to protect patients’ confidentiality. All experiments were performed in accordance with the guidelines of the Declaration of Helsinki and the institutional regulations.

### Classic culturing and susceptibility testing

The samples were cultured following methods routinely used in our institution. Briefly, samples were streaked onto five plates (Mediaproducts BV, Groningen, The Netherlands) - blood agar (aerobic), chocolate agar (aerobic), McConkey agar (aerobic), Brucella agar (anaerobic) and Sabouraud Dextrose + AV (aerobic) - and incubated overnight under aerobic and anaerobic atmosphere at 37 °C. The two pus samples were also plated onto Phenylethyl alcohol sheep blood agar (PEA), Kanamycin vancomycin laked blood (KVLB) agar and Bacteroides bile esculin (BBE) agar and incubated under anaerobic conditions overnight. The isolates recovered were subjected to susceptibility testing by Vitek 2 using either the AST-P559 (Gram-positive bacteria) or the AST-N344 (Gram-negative bacteria) card (bioMérieux, Marcy-l'Étoile, France) and identified by MALDI-TOF MS (Bruker Daltonik, Gmbh, Germany) using standard protocols.

### DNA extraction, library preparation and sequencing

The DNA for metagenomic sequencing was isolated using the Ultra-Deep Microbiome Prep (Molzym Life Science, Bremen, Germany), Micro-Dx™ kit (Molzym Life Science) or QIAamp DNA Microbiome Kit (Qiagen, Hilden, Germany) directly from the clinical samples and a negative control consisting of a mock sample of DNA and RNA free water (Table 1). These kits include human DNA depletion steps. The QIAamp DNA Microbiome Kit was used according to the manufacturer’s protocol with an additional 5 min air-dry step before elution. For microbial lysis, a Precellys 24 homogenizer (Bertin, Montigny-le-Bretonneux, France) set to 3 times 30 seconds at 5000 rpm separated by 30 seconds was used. After extraction, DNA was quantified with the Qubit 2.0 (Life Technologies, ThermoFisher Scientific, Waltham, Massachusetts, EUA) and NanoDrop 2000 (ThermoFisher Scientific). The DNA quality was assessed using the Genomic DNA ScreenTape and Agilent 2200 TapeStation System (Agilent Technologies, California, United States of America). Isolated DNA was purified using Agencourt AMPure XP beads (Beckman Coulter, California, United States of America) according to the manufacturer’s instructions, to eliminate small DNA fragments and chemical contaminants (e.g. benzonase). The DNA was then diluted to 0.2 ng/µl and 1 ng was used for the library preparation, using the Nextera XT Library Preparation kit (Illumina, California, United States of America), according to the manufacturer’s protocol. Cluster generation and sequencing were performed with the MiSeq Reagent Kit v2 500-cycles Paired-End in a MiSeq instrument (Illumina). Samples were sequenced in batches of 5 samples on a single flow cell.

For the DNA extraction of bacterial isolates (when an isolate was recovered from culture), we used the UltraClean Microbial DNA Isolation Kit (Mo Bio), with some modifications. We started with solid cultures and resuspended a 10 µl-loopfull of culture directly into the tube with the microbeads and microbead solution. The library preparation, cluster generation and sequencing was performed as described above. Strains were sequenced in batches of 12 to 16 on a single flow cell.

### Bioinformatics analyses

In order to evaluate and compare the accuracy and reliability of the bioinformatics analyses in providing the closest results to culture and WGS of any cultured isolates, three different pipelines (two commercially and one freely available) were used (Fig. 1). Different tools to perform raw read quality control, filtering and trimming were used and reads were mapped against the human genome (hg19) before performing taxonomic classification. Reads mapping to hg19 where removed from the analysis to increase the efficiency of the bioinformatics tools. Typing (MLST), phylogenetic analysis, plasmid analysis, detection of antimicrobial resistance and virulence genes was performed. To determine the appropriateness of SMg as predictor of the WGS (chromosome and plasmids), SMg results obtained were compared with the results of WGS of any bacterial isolates obtained from culturing the sample.

All the parameters used in each approach are available in Supplementary Table 1.

#### Unix-based approach

For the metagenomics data, read quality control and cleaning was performed using FastQC v0.11.5 and Trimmomatic v0.36, respectively, through the INNUca v2.6 pipeline (https://github.com/B-UMMI/INNUca), excluding assembly and polishing. Using a reference mapping approach against the human genome (UCSC hg19), human reads were discarded using Bowtie 2 v2.3.2^15^ and SAMtools v1.3.1^16^. Those paired reads that did not map against the human genome were used in subsequent analyses. The bacterial species were identified through Kraken v0.10.5-beta^17^ using the miniKraken database (pre-built 4 GB database constructed from complete bacterial, archaeal and viral genomes in RefSeq, as of Dec. 8, 2014), MIDAS^18^ using the midas_db_v1.2 database (>30,000 bacterial reference genomes, as of May 9, 2018) and MetaPhlAn2 v2.0^19^ using the database provided by the tool (~13,500 bacterial and archaeal, ~3,500 viral, and ~110 eukaryotic reference genomes, as of May 9, 2018). The sequence type (ST) was obtained through metaMLST v1.1^20^ based on the metamlstDB_2017. Antimicrobial resistance genes were detected using ReMatCh v3.2 (https://github.com/B-UMMI/ReMatCh), a read mapping tool that uses Bowtie 2 v2.3.2^15^ and the following rules for gene presence/absence: genes were considered present when ≥80% of the reference sequence was covered and the sample sequence was ≥70% identical to the one used as reference. For that, ResFinder database (2231 genes, downloaded on 29-06-2017) was used as reference and, due to the low coverage of microbial metagenomics samples, a minimal coverage depth of 1 read was set to consider a reference sequence position as covered (and therefore present in the sample data), as well as to perform base call (used for sequence identity determination). Finally, the assembly was accomplished through SPAdes v3.10.1^21^.

Plasmid detection was achieved by running the script PlasmidCoverage (https://github.com/tiagofilipe12/PlasmidCoverage), using the plasmid sequences downloaded from NCBI RefSeq (ftp://ftp.ncbi.nlm.nih.gov/genomes/refseq/plasmid/, as of May 11, 2017). The script uses Bowtie 2 v2.2.9^15^, to map the pre-processed input reads against the plasmid database (bowtie2 index for all plasmid sequences). For bowtie2 we used the ‘-k’ option, allowing each read to map to as many plasmid sequences as present in the NCBI RefSeq plasmid database (since plasmid sequences are modular)^22,23^. Then, this pipeline used SAMtools v1.3.1^16^ to estimate the coverage for each position, and reported the length of plasmid sequence covered (in percentage) and average depth (mean number of reads mapped against a given position in each plasmid). Plasmids with less than 80% of its length covered were excluded from the final results in line with what has described elsewhere^11^. The pATLAS tool (http://www.patlas.site) was used to visualize which plasmids were present.

For the WGS reads of the bacterial isolates, the whole INNUca v2.6 pipeline was run, including SPAdes assembly and polishing. Plasmids were detected as mentioned previously.

#### Commercial-based approach

The fastq files containing the reads were uploaded into CLC Genomics Workbench v10.1.1, using the following options: Illumina import, paired-reads, paired-end (forward-reverse) and minimum distance of 1 and a maximum distance of 1000 (default). The trimming was performed using the default settings, except the quality trimming score limit was set to 0.01 and we added a Trim adapter list containing Illumina adapters. The mapping was performed with the Map Reads to Reference tool, using the hg19 genome as reference. The default settings were used with the addition of the collect un-mapped reads option. The de novo assembly tool was used for the assembly (even for the metagenomics reads) and, apart from the word size, which was changed to 29, all the settings were default. Two tools were used for the microbial identification, Taxonomic Profiling and Find Best Matches using K-mer Spectra (Microbial Genomics Module). In both, the bacterial and fungal databases were downloaded from NCBI RefSeq (with the Only Complete Genomes option turned off; minimum length 500,000 nucleotides) on 08-07-2017 (bacterial, 70,868 sequences) and 25-05-2017 (fungal, 377 sequences). The antimicrobial resistance genes were detected, based on the assembled contigs, using the Find Resistance tool (Microbial Genomics Module) and were initially only considered present when they were ≥70% identical to the reference and ≥80% of the sequence was covered. The analysis was also repeated using ≥40% and ≥20% of sequence coverage for comparison purposes. The database containing the antimicrobial resistance genes was downloaded directly to the software from ResFinder (https://cge.cbs.dtu.dk/services/data.php, downloaded on 05-07-2017, 2156 sequences). The MLST was determined through the Identify MLST tool (Microbial Genomics Module), using all MLST schemes available at PubMLST (04-03-2017). The same database used for plasmid detection in Unix, was used for mapping the reads in CLC Genomics Workbench. Again, plasmids with less than 80% of its length covered were excluded from the final results. For WGS reads we used the Trim Sequences tool and the assembly, antimicrobial resistance genes detection, and MLST determination were performed as before.

#### Web-based approaches

The fastq files containing the reads were uploaded into the BaseSpace website. First, the raw forward and reverse fastq reads were subjected to FASTQ Toolkit for adapter/quality trimming and length filtering with standard settings and length filtering adjusted to a minimum of 100 and a maximum of 500. The trimmed reads were then used as input for all the following processes. The available microorganism identification apps Kraken v1.0.0, MetaPhlAn v1.0.0 and GENIUS v.1.1.0 were used with the standard settings/parameters. SEAR was used to detect antimicrobial resistance genes, maintaining the standard settings except for the clustering stringency which was set to 0.98 and the annotation stringency was set to 40. The SPAdes Genome Assembler v3.9.0 app was run with the standard parameters for multi cell data type. For metagenomic datatype settings, the running mode was set to only assembly and careful mode was disabled.

The reads were uploaded into CosmosID (https://app.cosmosid.com/login) and Taxonomer^24^ (https://www.taxonomer.com/) directly without any quality trimming. We used the Full Analysis mode in Taxonomer.

#### wgMLST analyses

Typing was done by MLST and wgMLST analyses obtained using Ridom SeqSphere + v4.0.1. The genomic data (assembled contigs) obtained from SMg was compared to the data obtained through WGS. Since no cg/wgMLST scheme was available for *Escherichia coli*, *Enterococcus faecalis*, *Ochrobactrum intermedium* and *Staphylococcus haemolyticus*, cgMLST and accessory genome schemes were constructed, using Ridom SeqSphere+ cgMLST Target Definer with the following parameters: a minimum length filter that removes all genes smaller than 50 bp; a start codon filter that discards all genes that contain no start codon at the beginning of the gene; a stop codon filter that discards all genes that contain no stop codon or more than one stop codon or that do not have the stop codon at the end of the gene; a homologous gene filter that discards all genes with fragments that occur in multiple copies within a genome (with identity of 90% and >100 bp overlap); and a gene overlap filter that discards the shorter gene from the cgMLST scheme if the two genes affected overlap >4 bp. The remaining genes were then used in a pairwise comparison using BLAST version 2.2.12 (parameters used were word size 11, mismatch penalty −1, match reward 1, gap open costs 5, and gap extension costs 2). All genes of the reference genome that were common in all query genomes with a sequence identity of ≥90% and 100% overlap and, with the default parameter stop codon percentage filter turned on, formed the final cgMLST scheme. The combination of all alleles in each strain formed an allelic profile that was used to generate minimum spanning trees using the parameter “pairwise ignore missing values” during distance calculation^25^.

### Statistical analysis

The sensitivity and positive predictive value of each taxonomic classification method were determined. Classical culture and MALDI-TOF identifications were considered as the gold standard. The true positives were considered when the same bacterial species were identified by culture/MALDI-TOF and the taxonomic classification method. The false positives were detected when bacterial species different from those identified by culture/MALDI-TOF, were identified by the taxonomic classification method. The false negatives were determined when the bacterial species identified by culture/MALDI-TOF were not identified by the taxonomic classification method.

### Accession codes

The paired-trimmed-un-mapped reads (hg19) generated for each sample have been submitted to SRA under project number SRP126380. The cgMLST schemes are deposited in figshare under the DOI:10.6084/m9.figshare.5679376.

## Electronic supplementary material

Supplementary Information
