# Author Correction: Critical steps in clinical shotgun metagenomics for the concomitant detection and typing of microbial pathogens

**DOI:** 10.1038/s41598-019-42134-9

**Published:** 2019-04-17

**Authors:** Natacha Couto, Leonard Schuele, Erwin C. Raangs, Miguel P. Machado, Catarina I. Mendes, Tiago F. Jesus, Monika Chlebowicz, Sigrid Rosema, Mário Ramirez, João A. Carriço, Ingo B. Autenrieth, Alex W. Friedrich, Silke Peter, John W. Rossen

**Affiliations:** 1 University of Groningen, University Medical Center Groningen, Department of Medical Microbiology, Groningen, The Netherlands; 2 Institute of Medical Microbiology and Hygiene, University of Tübingen, Tübingen, Germany; 3 Instituto de Microbiologia, Instituto de Medicina Molecular, Faculdade de Medicina, Universidade de Lisboa, Lisbon, Portugal

Correction to: *Scientific Reports* 10.1038/s41598-018-31873-w, published online 13 September 2018

This Article contains errors in Tables 2, 5 and 6.

In Table 2, the subheading ‘Kraken^b^’ should read ‘Kraken^bd^’

In Table 5, for the MetaPhlAn2 (Unix) method, the sensitivity and PPV values should read as 78% and 54% respectively. For the MIDAS (Unix) method, the sensitivity and PPV values should read as 78% and 21% respectively.

In Table 6, the subscript note ^a^ has been incorrectly included in the subheading ‘CLC Genomics Workbench^a^’, which should read ‘CLC Genomics Workbench’

