# Supplementary material for: Critical steps in clinical shotgun metagenomics for the concomitant detection and typing of microbial pathogens
Source: Sci Rep. 2018 Sep 13;8:13767. doi: 10.1038/s41598-018-31873-w (PMC6137123; doi:10.1038/s41598-018-31873-w)
Supplement: Supplementary file 1 — Supplementary Information [file 41598_2018_31873_MOESM1_ESM.pdf]

Critical steps in clinical shotgun metagenomics for the concomitant detection and typing of microbial pathogens

Natacha Couto[1], Leonard Schuele[1,2], Erwin C. Raangs[1], Miguel P. Machado[3], Catarina I. Mendes[1,3], Tiago F. Jesus[3], Monika Chlebowicz[1], Sigrid Rosema[1], Mário Ramirez[3], João A. Carriço[3], Ingo B. Autenrieth[2], Alex W. Friedrich[1], Silke Peter[2], John W. Rossen[1]

[1]University of Groningen, University Medical Center Groningen, Department of Medical Microbiology, Groningen, The Netherlands;
[2]Institute of Medical Microbiology and Hygiene, University of Tübingen, Germany;
[3]Instituto de Microbiologia, Instituto de Medicina Molecular, Faculdade de Medicina, Universidade de Lisboa, Portugal.

*Corresponding Author: Natacha Couto; Email: n.monge.gomes.do.couto@umcg.nl; Address:
University Medical Center Groningen, Department of Medical Microbiology EB80, Hanzeplein 1,
P.O. Box 30.001, 9700 RB Groningen, The Netherlands.

**FastQ Toolkit v2.2.0**	Done	
Minimum read length	32	
Sub-sampling	FALSE	
Adapter trim stringency	0.9	
Select respective adapters	TRUE	
Quality trimming	FALSE	
Poly-A/T Trimming	FALSE	
Read Filtering	FALSE	
Modify Reads	FALSE	
Fix Format	FALSE	
**FastQC v1.0.0**		
Kmer Size	5	
Use Conatminant Filter	TRUE	
**Kraken Metagenomics v1.0.0**		
Host Filter	TRUE	RefSeqhg19
Classification Database:	MiniKraken 20141208 (latest)	
Filter Threshold	0	
**Metaphlan v1.0.0**		
Sensitivity options for read-marker similarity (as descibed by BowTie2)	Very Sensitive	
**SPAdes Genome Assembler v3.9.0**		
Running Mode:	Error Correction & Assembly	
Dataset type	Multi Cell	
Careful Mode	Disable	
k-mer lengths	Auto	
**SEAR: Antibiotic Resistance v1.0.0**		
Read length cutoff (bases)	70	
Read quality score cutoff	20	
Read subtraction against E.coli reference genome (K12)?	No	
Clustering stringency (express % as a decimal)	0.98	
Annotation stringency (% length of reference ARG sequence mapped to by sequencing reads	40	
**GENIUS Metagenomics: Know Now v1.1.0**		
Can't set any settings in BaseSpace		

Trim Reads		
Trimmomatic v0.36 (INNUca v2.6 initial module)		
Quality trim	TRUE	
Phred Quality limit		5:20
Trim adapter list	Illumina adapters	
Remove 5' terminal nucleotides	TRUE	
Number of 5' terminal nucleotides		3
Remove 3' terminal nucleotides	TRUE	
Number of 3' terminal nucleotides		3
Discard short reads	TRUE	
Minimum number of nucleotides in reads		55
Map Reads to Reference		
Bowtie2 v2.3.2		
References	Homo sapiens (hg19) index	
Mode	end-to-end	
Mode option	sensitive	
Collect unmapped reads	TRUE	
Taxonomic classification		
Kraken v0.10.5-beta		
References	miniKraken database (Dec. 8, 2014)	
K-mer length		35
MIDAS		
References	midas_db_v1.2 (May 9, 2018)	
Word size for blast		28
Alignment coverage		0.75
MetaPhlAn2 v2.0		
References	default database (May 9, 2018)	
Minimum total nucleotide length for the markers		2000
Quantile value for robust average		0.1
Statistical approach for converting marker abundances into clade abundances	clade global	
Analysis type	profiling a metagenome in terms of relative abundance	
Identify MLST		
metaMLST v1.1		
References	metamlstDB_2017	
Bowtie2 mode	local	
Bowtie2 mode option	very sensitive local	
Collect unmapped reads	FALSE	
Search for and report all alignment	TRUE	
Find Resistance Genes		
ReMatCh v3.2		
References	ResFinder database (29-06-2017)	
Minimum coverage to consider a position as present		1
Minimum coverage depth to perform a basecall		1
Minimum gene coverage (%)		80
Minimum gene identity (%)		70
De novo assembly		
SPAdes v3.10.1		
Mode	careful	
Error correction	FALSE	
Read coverage cut-off value		2
List of K-mers	21,33,55,67,77	
Plasmid Detection		
Bowtie2 v2.3.2		
References	NCBI RefSeq (May 11, 2017)	
Mode	end-to-end	
Mode option	sensitive	
Collect unmapped reads	FALSE	
Multiple alignment	TRUE	

Illumina		
Discard sequence names	FALSE	
Discard quality scores	FALSE	
Selected files		
Paired-end reads	TRUE	
ReadOrientation	Forward Reverse	
minimum distance		1
maximum distance		1000
Remove failed reads	TRUE	
Quality score	NCBI/Sanger or Illumina Pipeline 1.8 and later	
MiSeq de-multiplexing	FALSE	
Illumina trim	FALSE	
Trim Reads		
Quality trim	TRUE	
Quality limit		0.05
Ambiguous trim	TRUE	
Ambiguous limit		2
Trim adapter list	Illumina adapters	
Use colorspace	FALSE	
Remove 5' terminal nucleotides	FALSE	
Number of 5' terminal nucleotides		1
Remove 3' terminal nucleotides	FALSE	
Number of 3' terminal nucleotides		1
Discard short reads	TRUE	
Minimum number of nucleotides in reads		30
Discard long reads	FALSE	
Maximum number of nucleotides in reads		1000
Map Reads to Reference		
References	Homo sapiens (hg19) sequence	
Masking mode	No masking	
Masking track		
Match score		1
Mismatch cost		2
Cost of insertions and deletions	Linear gap cost	
Insertion cost		3
Deletion cost		3
Insertion open cost		6
Insertion extend cost		1
Deletion open cost		6
Deletion extend cost		1
Length fraction		0.5
Similarity fraction		0.8
Global alignment	FALSE	
Color space alignment	TRUE	
Color error cost		3
Auto-detect paired distances	TRUE	
Non-specific match handling	Map randomly	
Find Best Matches using K-mer Spectra		

References	NCBI references (2017-07-08)	
K-mer length		16
Only index k-mers with prefix	ATGAC	
Check for low quality and contamination	TRUE	
Fraction of unmapped reads for quality check		0.1
De Novo Assembly		
Mapping mode	Create simple contig sequences (fast)	
Update contigs	TRUE	
Mismatch cost		2
Insertion cost		3
Deletion cost		3
Colorspace error cost		3
Length fraction		0.5
Similarity fraction		0.8
Colorspace alignment	TRUE	
Alignment mode	local	
Match mode	random	
Create list of un-mapped reads	FALSE	
Automatic bubble size	TRUE	
Bubble size		50
Automatic word size	TRUE	
Word size		20
Minimum contig length		200
Guidance only reads		
Perform scaffolding	TRUE	
Auto-detect paired distances	TRUE	
Create report	TRUE	
Find Resistance		
DB	Database for Find Resistance (2018-02-02)	
Minimum identity %		70
Minimum length %		20
Filter overlaps	TRUE	
Local Realignment		
Realign unaligned ends	TRUE	
Multi-pass realignment		2
Guidance-variant track		
Maximum guidance-variant length		100
Force realignment to guidance-variants	FALSE	
InDels and Structural Variants (2)		
P-Value threshold		1.00E-04
Maximum number of mismatches		3
Ignore broken pairs	TRUE	
Filter variants	FALSE	
Minimum number of reads		2
Minimum relative consensus coverage		0
Minimum quality score		0
Restrict calling to target regions		

Local Realignment (2)		
Realign unaligned ends	TRUE	
Multi-pass realignment		2
Guidance-variant track	Defined by: InDels and Structural Variants (2)	
Maximum guidance-variant length		100
Force realignment to guidance-variants	FALSE	
Identify MLST Scheme from Genomes		
Schemes	PubMLST (04-03-2017)	
Identify MLST		
Scheme	Defined by: Identify MLST Scheme from Genomes	
Low coverage reported when below		30
